# Synthesis, antimicrobial, antioxidant studies of thermally stable nanocomposite films doped with CoO-NPs for active food packaging applications

**DOI:** 10.1371/journal.pone.0338180

**Published:** 2025-12-05

**Authors:** Maqam Ali, Muhammad Kaleem Khosa, Awal Noor, Sadaf Qayyum

**Affiliations:** 1 Department of Chemistry, Government College University Faisalabad, Faisalabad, Pakistan,; 2 Department of Chemistry, College of Science, King Faisal University, Al-Hassa, Saudi Arabia; National Research Centre (NRC), EGYPT

## Abstract

This research work presents a fabrication and study to explore different features of Gelatin/ Polyethylene glycol/ Chitosan ternary composite films doped with cobalt oxide nanoparticles. Such films were prepared by solution casting technique coupled with sonication with varying concentrations of CoO-NPs (0.5%–2.5%). Multiple techniques like, FT-IR, XRD, SEM, TGA and DMA were used to characterize and test their stability. FT-IR and XRD confirmed the cross-linking among the biopolymers and metal oxide nanoparticles. Smooth surface morphology was observed by SEM while TGA and DMA was used to determine the thermal stability and flexibility of nanocomposites. The flexibility (EB) and tensile strength of the GPC blend was found to be considerably increased by the addition of CoO-NPs up to 2.5%. It has been also observed that as the concentration CoO-NPs in gelatin/polyethylene glycol/Chitosan, increased (up to 2.5%), the nature of GPC composites films changed from hydrophilic to hydrophobic After the characterization, the synthetic nanocomposites films were subjected to antibacterial and antioxidant analysis. The nanocomposites films showed significant activity against *S. aureus* and *E. coli*, and were further applied to antibiofilms formation activity. Results showed that, increased thermal stability and flexibility, reduced moisture absorbance, water permeability, good antibacterial, antioxidant and antibiofilm activities against foodborne pathogens make them good choice for active food packaging applications.

## Introduction

The food packaging sector is looking for more sustainable options as a result of growing environmental concerns over synthetic packaging materials [1]. Biopolymers are well known for their significant contributions in various fields, particularly in pharmaceuticals, textiles, and environmental sustainability [2]. Their distinctive features like biocompatibility and biodegradability make them essential in developing innovative solutions like utilizing in food packaging, wound healing, providing moist environments that enhance tissue repair [3]. Their compatibility with biological systems allows for minimal immunogenicity in medical applications [4] reducing plastic waste and for active food packaging material as they are derived from renewable sources and biodegradable [5]. Gelatin has biological and photocatalytic applications due to its biocompatibility, structural properties, and ability to enhance catalytic efficiency [6]. Its incorporation into various nanocomposites and biocatalysts has shown promising results across the multiple studies like it effectively supports for enzyme immobilization, enhancing the stability and activity of biocatalysts like laccase, which is vital for wastewater treatment [7]. Chitosan is recognized as a nontoxic, non-antigenic, and biocompatible polymer, known for its bio adhesive, wound-healing, and antimicrobial properties [8]. Polyethylene glycol (PEG), plays a key role to improve the performance of different nanomaterials for biological and photocatalytic applications [9]. Its ability to modify surface properties significantly improves stability, biocompatibility, and catalytic efficiency. Polyethylene glycol functionalization reduces the agglomeration in nanoparticles and increases the photocatalytic activity and antimicrobial properties of various nanoparticles [10,11]. Similarly, Polyethylene Glycol-modified zinc titanate and cobalt oxide nanocomposites exhibited improved photocatalytic degradation rates for organic pollutants, with significant hydroxyl radical production [12]. Previous studies mainly explored ZnO, CuO, or TiO₂ in simple binary polymer systems, often focusing on limited properties, whereas CoO-NPs offer distinct advantages for their multifunctional biological applications [13–15]. CoO shows stronger redox activity and generates reactive oxygen species that enhance antibacterial and antibiofilm effects. Their unique properties, such as biocompatibility make them suitable for various environmental and biomedical applications as demonstrated by their use in a chitosan-coated nanocomposite [16,17]. Their synthetic methods, especially, green synthesis methods using plant extracts enhance the biocompatibility and functional properties of Cobalt oxide nanoparticles, making them suitable for medical applications [18].

The aim of this research is to fills the gap previous and current studies by introducing CoO into a ternary gelatin/polyethylene glycol/ chitosan (GPC) matrix and tune the properties of GPC blends doped with Cobalt oxide nanoparticles. The effect of CoO-Nps on performance of GPC films was investigated by thickness films, morphology, structure, thermal stability and flexibility. After characterization, all films were subjected to antibacterial, antioxidant and antibiofilm activities against foodborne pathogens. The effect of CoO-Nps on performance of GPC films was investigated by thickness films, morphology, structure, thermal stability and flexibility, antibacterial, antioxidant properties, antibiofilm activity.

## Materials and methodology

### Chemicals

Chitosan powder (Mol. Wt. 30 KD), Laboratory-grade gelatin (Type B) derived from bovine skin, polyethylene glycol (PEG) with a molecular weight of 8,000, cobalt chloride hexahydrate (CoCl_2_·6H_2_O), glacial acetic acid (CH_3_COOH) and sodium hydroxide (NaOH) were analytical grade, procured from Sigma-Aldrich, Pakistan and used as received.

### Characterization

The structure of synthesized GPC-CoO nanocomposite films was analyzed by fourier transform infrared spectrophotometer (FT-IR, Perkin Elmer, Bruker) within the range of 4000–400 cm^-1^. crystallinity and structural phase of material was determined by X-ray diffractometer (Bruker D8 Advance), with Cu-Kα radiation (wavelength = 1.5405 Å) under conditions of 30 kV and 40 mA at a scanning rate of 5° min ⁻ ¹. Mechanical properties were assessed using a dynamic mechanical analyzer (DMA Q800 V21.3 Build 96). nano-sized surface modification was examined through scanning electron microscopy (SEM model FEI-NOVA Nano SEM-450). Thermal stability was determined by thermogravimetric analysis (Perkin Elmer TGA 4000) at heating rate of 5 ^0^C per minute in nitrogen atmosphere over a temperature range of 50–700 ^0^C. The optical properties of the prepared films were recorded using a double beam UV-Visible spectrophotometer (Stalwart SAT-8200 Series), measurements were taken in the wavelength range of 200–800 nm, with an accuracy of ± 0.2 nm. Hand-held micrometer was used to measure thickness of films. (Dial Thickness Gauge 7301, Mitutoyo Corporation, Kanagawa, Japan)

### Preparation of Cobalt oxide NPs

CoO-NPs (size: 20 nm) were prepared by co-precipitation process as described in literature with slight modifications [19]. For this purpose, 100 mL of cobalt chloride hexahydrate (1.0M) solution in deionized (DI) water. To this solution, an aqueous solution of sodium hydroxide (1M) was added dropwise until pH of solution reaches 9. The resulting mixture was continuously stirred about half an hour to ensure thorough mixing. Subsequently, the precipitates were filtered, washed thrice with water then dried. Once dried, the powder was calcinated at 500°C for 4 hr.

### Fabrication of Gelatin/Polyethylene Glycol/Chitosan/CoO) nanocomposites (GPC/CoO)

GPC/CoO nanocomposites films were prepared by solution casting method followed by ultrasonic process with slight modification [20]. Gelatin, Polyethylene Glycol, and Chitosan solutions (1:1:1) were stirred vigorously to ensure complete mixing at ambient temperature. The resulting blend was designated as GPC solution. To this solution, 0.5, 1.0, 1.5, 2.0, and 2.5% w/w of cobalt oxide nanoparticles (CoO-Nps) were mixed via ultrasonic blending, followed by filtration. The GPC/CoO-nanocomposite films were casted onto glass petri dishes and dried at 50°C in oven about 24 hr.

### Water vapor permeability test

The water vapor permeability (WVP) GPC blends and GPC blend doped with CoO nanoparticles was conducted gravimetrically using standardized procedures to ensure reliable and reproducible results [21,22]. Prepared films of size, 5 × 5 cm^2^ were dried at 100 °C in oven. After drying, films were horizontally mounted on top of WVP measuring cups filled with 15 mL water. The sealed assembly was weighted and placed in an oven for 24 h at 50 °C. Subsequently, the cups were weighted after every 30 mint and the change in weight was noted as a function of time. The weight loss of the cups was plotted against time. From the slope, Water vapor transfer rate (g/m^2^. s) was calculated and put the value of WVTR in following equation to determine the WVP (g/ m²/24hrs).


$$\text{WVP}=\frac{\text{WVTR }\times \text{ L}}{\Delta \text{P}}$$

where L indicates the film thickness (mm), and ΔP corresponds to the water vapor pressure difference at 50 °C, which is 12.344 kPa.

### Moisture absorption

To evaluate the moisture absorption (MA) of the films, films were cut into (5 x 5) cm^2^ dimensions, weighted (W_0_) and dried in oven for 24 h at 100 ºC [23]. After that films were weighted again (W_t_). Moisture absorption was calculated using the formula:


$$\text{MA }(\%\;)=\frac{\;{\text{W}}_{\text{t}}-\;{\text{W}}_{\text{0}}}{{\text{W}}_{\text{0}}}\text{ x 100}$$

where W_t_: final weight of the sample, and W_0_: initial weight. This method provided an accurate measure of the films’ capacity to absorb moisture under controlled conditions.

### Water solubility test

Water solubility test was performed as per literature method [24]. typically, film was cut into dimensions of (5 × 5 cm²) and oven dried at 100 °C to get constant weight (m_0_). After drying, film was put glass beaker with100 mL of water for 24 h. The moist film was dried under the same conditions until the weight remains constant. Water solubility of nanocomposite film was calculated as.


$$\text{WS }(\%\;)=\frac{\;{\text{m}}_{\text{0}}-\;{\text{m}}_{\text{1}}}{{\text{m}}_{\text{0}}}\text{ x 100}$$

where: m_0_ is weight of film before immersion, and m_1_ is the weight of the films after immersion and subsequent drying. This method allowed for a precise evaluation of the film solubility in water, contributing to the overall assessment of their water resistance properties [21].

### Antibacterial assay

Antibacterial activity of prepared nanocomposite films was investigated by “Disc diffusion method” [25] against three food pathogens: Gram-negative bacterial strains including *Escherichia coli* (ATCC*-*8739), *Pseudomonas aeruginosa* (ATCC-9027), and *Salmonella Typhi* (ATCC-6539), and the Gram-positive Bacteria *Bacillus subtilis* (ATCC 6633), *Staphylococcus aureus* (ATCC-6538, and *Streptococcus pyogenes* (ATCC-12344). GPC film and GPC/CoO-nanocomposite solution in DMSO (20 mg/mL) was added on agar plates (Mueller Hinton), which has 2 ml of inoculum containing 10^6^ colony-forming units (CFU/ml) of bacterial strains under investigation. The plates were incubated for 24 h at 37ºC and examined zones of inhibition (mm). To determine an average zone of inhibition, each measurement was run in triplicate and results were compared with zone of inhibition of Cefixime, a standard antibacterial drug.

### Radical scavenging assay

The free radical scavenging activity of nanocomposite films was evaluated using 2,2-diphenyl-1-picryl-hydrazyl (DPPH) assay with slight changes [26]. A stock solution of each film in DMSO was prepared (5 mg/mL). The 0.1 mM of DPPH solution in methanol (2980 µL) was added to 20 µL of concentration of film’s solution (5, 10, 20, 40, 100, and 200 μg/mL). Reaction mixture was incubated at 37°C in dark for 30 minutes. The color of reaction mixture was gradually changed to pale yellow. After change in colour, absorbance was measured at 517nm by UV/Visible spectrophotometer. Each experiment was carried out in triplicate under same conditions. Ascorbic acid (5 mg/mL) was used a reference. The percentage of radical scavenging was calculated by following equation.


$$\%\;scavenging\;activity=\frac{absorbance\;of\;control-absorbance\;of\;test\;sample}{absorbance\;of\;control}x100$$

### Antibiofilm assay

The effectiveness of GPC/CoO nanocomposites in preventing biofilm formation was assessed using *S. aureus* and *E. coli* as model pathogens, revealing a range of antibacterial activity from high to low. The biofilm forming bacteria were cultured in 10 mL of tryptic soy broth (TSB) with 1% glucose at 37°C for 24 h. After that, 200 μL bacterial strain was introduced to 96-well microtiter plates that contained 20 μg/mL of GPC/CoO nanocomposite, and the plates were subsequently incubated for about 24 h at 37°C. After that, wells were washed with a buffer solution of pH 7.2 (saline phosphate) thrice, then dried at room temperature. crystal violet solution (200 μL, 50%,) was added and plates were incubated at 37°C for twenty minutes. The excess of stain was removed by washing with water followed by ethanol. and dried. Absorbance of crystal violet solution was noted at 490 nm by microplate reader. Biofilm % inhibition was noted. Cefixime (25 g mL ⁻ ¹) was used as a standard drug [27,28]. Percentage of biofilm inhibition was measured as:


$$\%\;inhibition=1-(\frac{optical\;density\;of\;test\;sample}{optical\;density\;of\;negative\;control})\times 100\mathrm{\backslash ]}$$

### Statistical analysis

Each experiment was performed in triplicate, where n = 3 and the data was expressed as mean ± standard deviation (SD). The obtained data was statistically validated through the use of Minitab Software’s ANOVA. Each experiment was carried out in triplicate. where level of least significant difference was set at p < 0.05.

## Results and discussion

### FT-IR analysis

**Fig 1:**
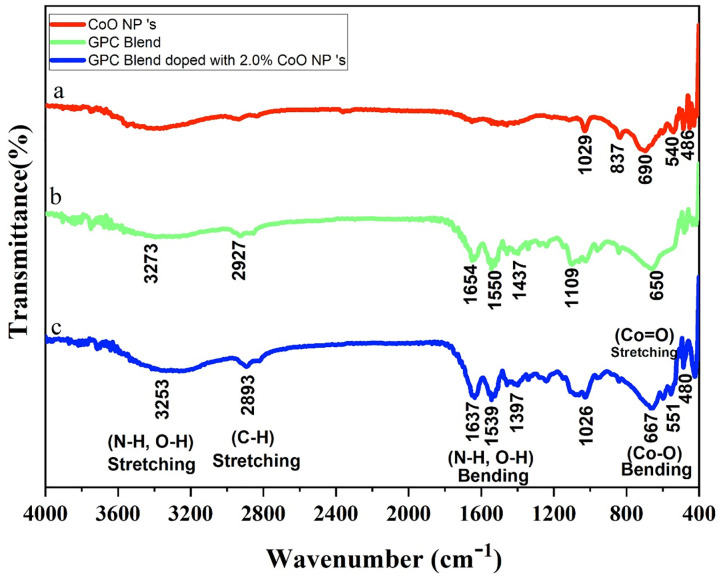
FT-IR spectra of a) CoO-NPs b) GPC blend c) GPC/2% CoO- nanocomposite.

Different functional groups in cobalt oxide-NPS, GPC films, and GPC/CoO-nanocomposites were assessed by FT-IR as shown in Fig 1 (a, b and c). Three prominent peaks at 690 cm ⁻ ¹, 540 cm ⁻ ¹, and 486 cm^-1^, corresponding to O-Co-O and Co-O stretching vibrations (Fig 1a). confirmed the formation of CoO-NPs, [29]. The cross-linking between gelatin, polyethylene glycol, chitosan and CoO-Nps identified by slight changes in the intensity and shifts in the position of certain characteristic bands of gelatin, polyethylene glycol, and chitosan in GPC/CoO nanocomposites, from 3273 cm^-1^ (O–H), 1654 cm^-1^ (CO-stretching), 1550 cm^-1^ (N-H-stretching), 1437 cm^-1^ (COO) _sym_, 1109 cm^-1^ (C-O-C stretching) to 3253 cm^-1^, 2893 cm^-1^, 1637 cm^-1^, 1539 cm^-1^, 1397 cm^-1^, 1026 cm^-1^ respectively (Fig 1b & c). Appearance of new intense peaks at 551 cm^-1^ and 480 cm^-1^ in GPC/CoO-NPs, confirmed the homogenous mixing of GPC blend with CoO-NPs [30].

### XRD analysis

**Fig 2:**
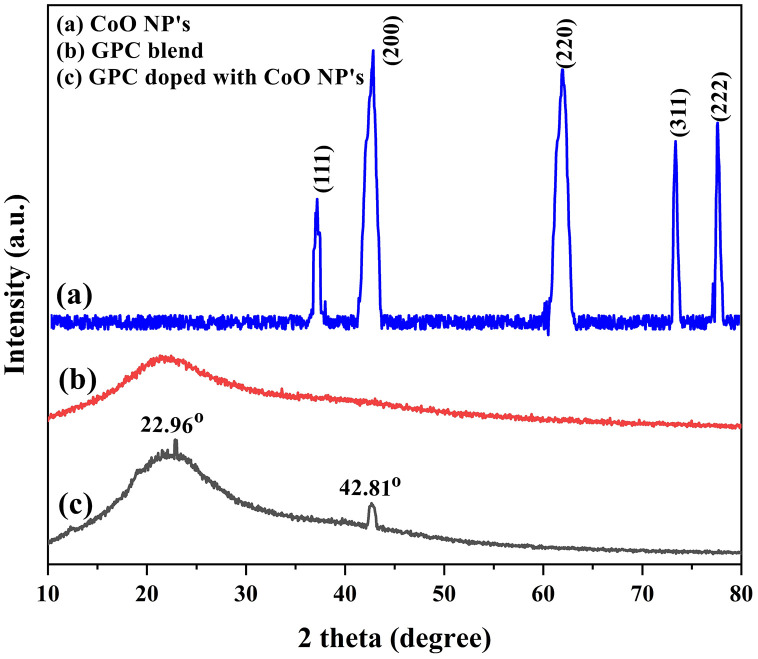
XRD Pattern of a) CoO-NPs b) GPC blend c) GPC/2.5% CoO nanocomposite.


XRD data of CoO-NPs, GPC blend and GPC/CoO-nanocomposite is shown in Fig 2. Fig 2(a) illustrates the XRD pattern of CoO-NPs synthesized via the co-precipitation method. The high-intensity peaks at 37.01°, 42.47°, 61.81°, 73.34°, and 77.65° are the diffraction planes (111), (200), (220), (311), and (220), respectively. These 2θ values align with the reference data from the International Centre for Diffraction Data (ICDD Code No. 00-001-1227), confirming the phase of CoO [31]. The crystallite size of CoO-NPs was calculated using the Debye–Scherrer equation:

$$D=\frac{\text{0}.\text{9}\lambda }{\beta \text{cos}\theta }$$

Here, D is the average size of the NPs, λ is the wavelength of Cu-Kα radiation, β is the full width at half maximum (FWHM) in radians, and θ is the diffraction angle in radians. The CoO nanoparticle size distribution ranged between 7 and 35 nm with an average size of 18.05 nm. (Table 1). Fig 2(b) presents the XRD pattern of a Gelatin/Polyethylene glycol/Chitosan (GPC) blend, which does not exhibit any distinct crystalline peaks, suggesting the amorphous nature of the composite. In contrast, as shown in Fig 2(c), the addition of CoO-NPs into the GPC blend resulted in the appearance of peaks at 22.96° and 42.81°, signifying an increase in crystallinity. This indicates that the GPC blend effectively interacts with CoO-NPs, leading to the formation of a nanocomposite with enhanced crystallinity [32].

**Table 1:** The peak positions of X-ray Diffraction of CoO NPs.

Peak Position 2θ (Degree)	*FWHM β (Degree)	Crystallite Size (nm)
37.0109	0.4965	17.62
42.4664	1.2552	7.09
61.8132	1.2682	7.63
73.341	0.4591	22.53
77.6469	0.3011	35.37
**Average crystallite size (nm)**		**18.05 nm**

* FWHM: Full-Width Half Maximum.

### SEM study

**Fig 3:**
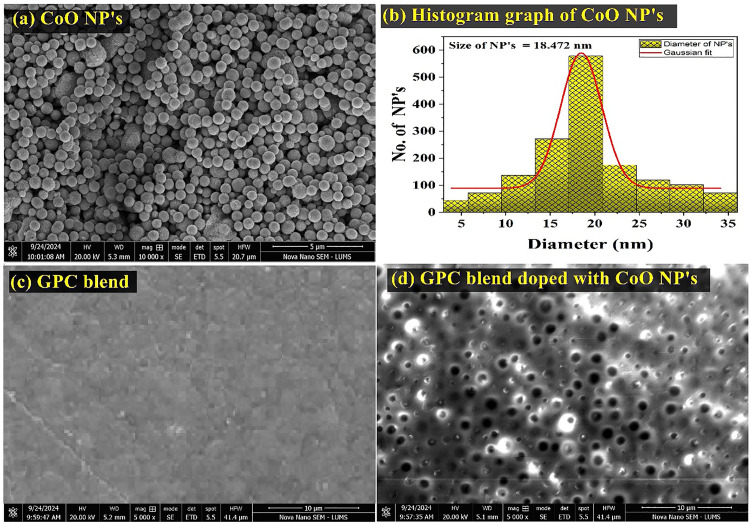
SEM images of CoO-NPs and GPC/2.5% CoO nanocomposite.

The morphology of cobalt oxide NPs, GPC blend, and GPC blend doped with cobalt oxide NPs was analyzed by scanning electron microscopy, as shown in Fig 3 (a-d). Results showed that CoO-NPs are crystalline with small spherical, relatively uniform and separate morphology, as depicted in Fig 3a. The average crystallite size was calculated by using Image J. software (18.47 nm), which was comparable as calculated by the Debye-Scherrer method (18.05 nm). The particle size histogram depicted in Fig 3(b) illustrated the fitting of the data using a Gaussian function, which was derived from the SEM measurements. The smooth surface of gelatin/polyethylene glycol/chitosan (GPC) film as revealed in Fig 3c confirmed the homogenous mixing and chemical bonding among polymers. CoO-NPs are uniformly distributed in GPC films without aggregation up to 2.5%, showing strong interaction between the CoO-NPs and GPC blend and changed the properties of GPC/ CoO-nanocomposites from hydrophilic to hydrophobic [33].

### Thermal analysis

**Fig 4:**
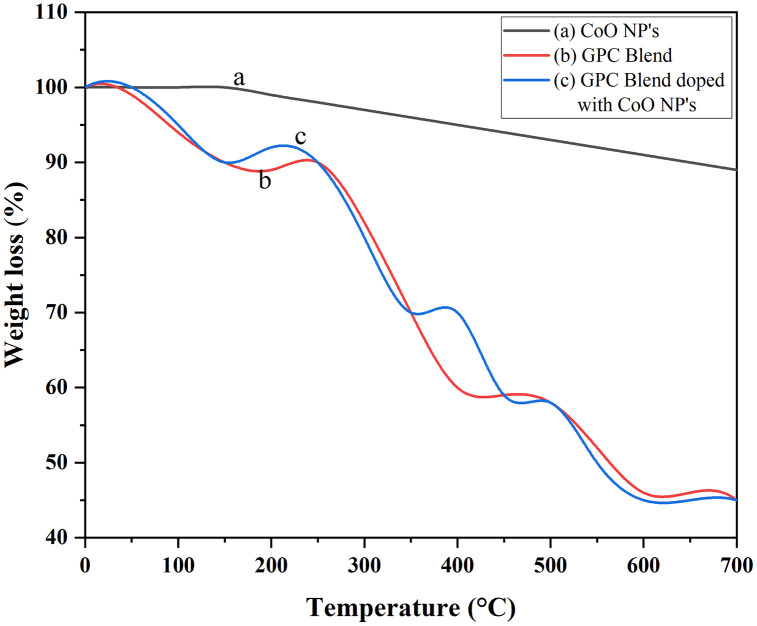
TGA of CoO-NPs and GPC/ 2.5% CoO nanocomposite.

Thermal stability of nanocomposite films is an important property for their application in food packaging. The thermogravimetric analysis (TGA) curves of pure CoO nanoparticles, Gelatin/Polyethylene Glycol/Chitosan (GPC) blends and GPC films doped with CoO nanoparticles are presented in Fig 4 (a, b, c). CoO nanoparticles show no weight loss curve as it has no volatile component in it. The decomposition of GPC blends takes place in three steps. i) first step, at 60–90 °C, corresponds to moisture removal. ii) second step, at 200–300 °C, reflects the decomposition of linkages among the Gelatin/ Polyethylene Glycol/ Chitosan molecules and iii) final step, at 400–550 °C, represents the breakdown of residual materials [30]. The TGA results demonstrate that GPC blends doped with CoO nanoparticles exhibit higher thermal stability compared to the neat GPC blend. The final stage, above 500 °C, reflects the rapid decomposition of components such as gelatin, polyethylene glycol, and chitosan. The inclusion of CoO nanoparticles slightly increases the thermal stability of nanocomposites. This enhanced thermal stability indicates that these films are suitable for applications in food packaging [31,32].

### Mechanical properties

**Fig 5:**
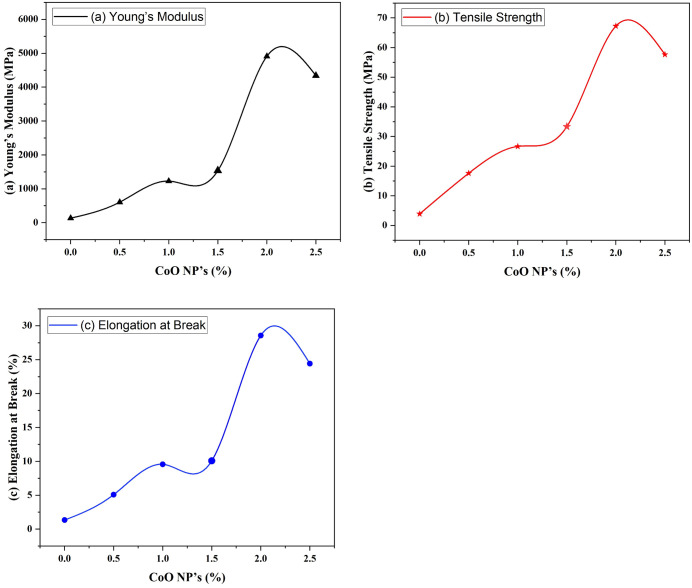
CoO-NPs and GPC/ 2.5% CoO nanocomposite a) Young’s Modulus b) Tensile strength c) % Elongation at break.

The flexibility and mechanical strength of composite films is an important feature in assessing their ability to maintain integrity under challenging environmental circumstances, including those that could arise during packing [33]. The flexibility (EB) and tensile strength of the GPC blend was found to be considerably increased by the addition of CoO-NPs up to 2.5%. However, at higher concentration of CoO-NPs (>2.5%), a decrease in tensile strength and elongation at break was observed as shown in Figs 5 (a), (b) and (c). Agglomeration and uneven distribution of nanoparticles in GPC blend, create a voids within the GPC matrix; thereby, a decrease of 57.66 MPa for tensile strength and 24.42% for elongation at break was observed. Furthermore, CoO-NPs is expected to influence the thermal and barrier properties of the GPC films, which are critical for applications such as packaging materials. The interactions between the nanoparticles and the polymer matrix could reduce the free volume within the films, potentially decreasing their permeability to gases and moisture. This characteristic enhances the suitability of the films for protective applications, where both mechanical strength and barrier properties are essential. Future research may focus on exploring these additional properties to fully understand the comprehensive benefits of CoO-NPs in polymer composite films [34].

### Water resistance properties

#### Water vapor permeability.

The water vapor permeability (WVP) test is a fundamental method for assessing the ability of nanocomposite films to resist the passage of water vapor. This property is particularly significant in determining the durability and functionality of films in various applications, including food packaging, where moisture resistance plays a crucial role in extending the shelf life of food products. Table 3 summarizes the WVP results for the analyzed film samples, which include Gelatin/ Polyethylene Glycol/ Chitosan (GPC) blends and GPC doped with CoO nanoparticles (CoO NPs) at varying concentrations (0.5%, 1.0%, 1.5%, 2.0%, and 2.5%). The findings indicate that GPC films doped with CoO-NPs exhibit lower moisture permeability compared to the undoped GPC blend films. Notably, the incorporation of CoO-NPs up to 2.5% significantly reduced WVP, reflecting an improvement in the film’s barrier properties. This trend attributed to the enhancement of the composite structure’s compactness due to improved hydrogen bonding between polymer components at lower nanoparticle concentrations. In contrast, higher concentrations of CoO-NPs (ca > 2.5%), an increase in WVP was observed due to fact that at higher concentration of CoO-NPs leads to agglomeration and uneven distribution, which created a voids within the GPC matrix, and increased the water vapor permeability of film [18].

**Table 2:** Water resistance properties of *GPC/CoO-nanocomposite films.

Components	Water Vapor Permeability (g/m^2^/24h)	Moisture absorption (%)	Water solubility (%)	Thickness (mm)
GPC Blend	0.84	10.28	44.02	0.05
Blend:0.5% CoO NP’s	0.62	8.29	32.18	0.05
Blend:1.0% CoO NP’s	0.58	7.07	28.39	0.05
Blend:1.5% CoO NP’s	0.46	4.64	24.67	0.05
Blend:2.0% CoO NP’s	0.32	3.35	21.02	0.05
Blend:2.5% CoO NP’s	0.30	3.29	18.11	0.05
Blend:3.0% CoO NP’s	0.41	3,81	22.42	0.05

*GPC; Gelatin/ Polyethylene Glycol/ Chitosan.

**Table 3:** Antibacterial activity of CoO-NPs, GPC blend and GPC/CoO nanocomposites (*invitro*).

Name of films	Zone of Inhibition (mm)					
	*B. subtilis*	*S. aureus*	*S. pyogenes*	*E. Coli*	*P. aeruginosa*	*S. Typhi*
CoO-NPs	20 ± 1	17 ± 1	11 ± 1	18 ± 1	10 ± 1	16 ± 1
GPC	16 ± 1	21 ± 1	13 ± 1	16 ± 1	13 ± 1	14 ± 1
GPC + 0.5% CoO-NPs	18 ± 1	21 ± 1	15 ± 1	19 ± 1	13 ± 1	17 ± 1
GPC + 1.0% CoO-NPs	19 ± 1	22 ± 1	17 ± 1	23 ± 1	15 ± 1	20 ± 1
GPC + 1.5% CoO-NPs	22 ± 1	25 ± 1	19 ± 1	23 ± 1	19 ± 1	22 ± 1
GPC + 2.0% CoO-NPs	24 ± 1	26 ± 1	23 ± 1	25 ± 1	20 ± 1	24 ± 1
GPC + 2.5% CoO-NPs	25 ± 1	30 ± 1	25 ± 1	28 ± 1	23 ± 1	25 ± 1
Cefixime	33 ± 1.5	31 ± 1	35 ± 1.5	29 ± 0.5	36 ± 1.25	31 ± 2

GPC = Gelatin/ Polyethylene glycol/ Chitosan.

Activity (zone of inhibition): 10-15 mm = poor activity, 16-25mm = good activity, 26 - 40mm = strong activity.

### Moisture absorption

Moisture absorption (MA) is a key feature of nanocomposite films for food packaging, as lower moisture absorption enhances the reliability of these films for food preservation. Recent advancements in biopolymer-based nanocomposite films have demonstrated significant improvements in moisture absorption properties. Table 3 presents the MA test results for Gelatin/ Polyethylene Glycol/ Chitosan (GPC) blends and GPC films doped with CoO nanoparticles (CoO-NPs) at various concentrations (0.5%, 1.0%, 1.5%, 2.0%, and 2.5%). The data reveals that the GPC blend exhibits the higher moisture absorption value, 10.28% after one week, whereas the nanocomposite films containing CoO-NPs have lower MA. This reduction in hydrophilic feature could be due to the filling of cavities within GPC films by strong interaction of CoO-nanoparticles components of GPC [19].

### Water solubility

Biocompatible polymers utilized in food packaging require minimal water solubility. to evaluate this property. A common limitations of biopolymers are, the higher moisture sensitivity compared to synthetic polymers, prompting recent research to focus on enhancing their water resistance. The WS results for Gelatin/ Polyethylene Glycol/ Chitosan (GPC) blends and GPC films doped with varying concentrations of CoO nanoparticles (0.5%, 1.0%, 1.5%, 2.0%, and 2.5%) are presented in Table 2. The addition CoO-NPs up to 2.5% w/w, significantly reduced WS compared to neat GPC films, indicating improved water resistance, however, at higher concentrations (above 2.5% CoO-NPs) the WS increased again due to the accumulation of CoO-NPs which reduce hydrogen bonding between the GPC blend and CoO-NPs, [18].

### UV-visible spectroscopy analysis

**Fig 6:**
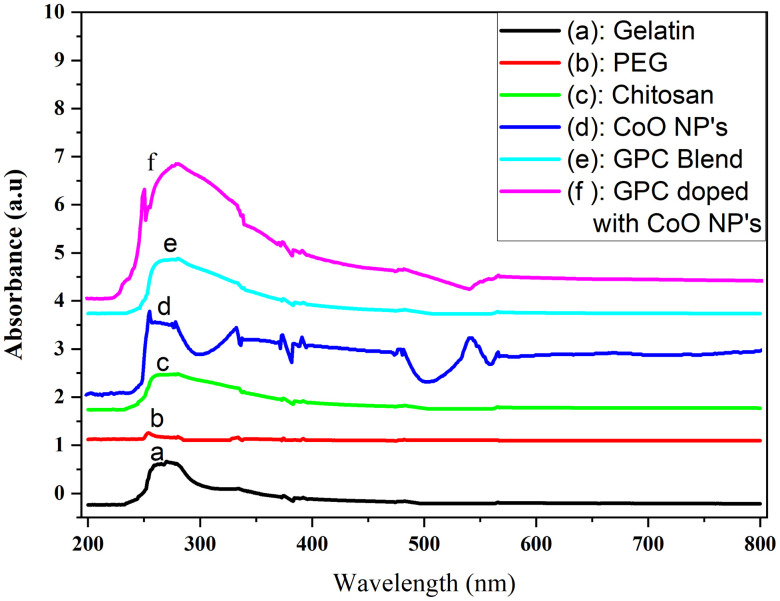
UV/visible spectra of a) Gelatin b) Polyethylene Glycol c) Chitosan d) CoO-NPs e) GPC blend and f) GPC/2.5% CoO nanocomposite.

UV-Visible spectroscopic analysis was carried out to measure the optical properties and interaction among the components of prepared materials at different wavelengths, The UV-Visible absorption spectra of pure Gelatin, Polyethylene Glycol, Chitosan and GPC/ CoO nanocomposite are shown in Fig 6(a-f). The pure gelatin exhibited a strong absorbance peak in the UV region around ~270 nm, which is due to presence of amino groups in proline, argine and glycine (Fig 6 a). Polyethylene Glycol being a non-aromatic and non-conjugated polymer, having no chromophores showed negligible absorbance throughout the scanned wavelength range as in Fig 6 b. Chitosan (Fig c) also showed a prominent peak near ~270 nm due to its polycationic nature and π-π* transition of C = O group of chitosan capable of absorbing UV radiation [35]. The CoO NPs (Fig 6d) displayed a distinct absorption band around ~310 nm, attributed to d-d transitions of Co² ⁺ ions which indicating their characteristic surface plasmon resonance and confirming the successful synthesis of the nanoparticles. When gelatin, Polyethylene Glycol, and chitosan were blended (Fig 6 e), a notable increase in absorbance was observed compared to individual components, suggesting possible intermolecular interactions and improved light-harvesting ability due to the composite structure. The GPC blend doped with CoO NPs (Fig 6 f) revealed a significant enhancement in absorbance peak intensity, particularly in the UV region (200–400 nm), with a broad peak around ~280–320 nm. This increase can be attributed to the synergistic interaction between the polymeric matrix and CoO nanoparticles, resulting in enhanced optical properties. The extended tail in the visible region up to 600 nm in the doped sample also suggests increased light absorption and possible electronic transitions due to incorporation of CoO-NPs and can be used for active food packaging applications [36].

### Antibacterial activity

The nanoparticle has a good penetration power and kill the microbes in host molecules [37] Chitosan, when combined with gelatin and/or polyethylene glycol mostly demonstrates antibacterial activity by electrostatically rupturing of microbial cell membranes and changing their permeability, which causes intracellular contents to seep out, causing cell death [38]. The antibacterial screening results are presented in Table 3. The antibacterial activity of GPC films and GPC/CoO nanocomposite films was compared with a standard drug (Cefixime), and found that GPC/CoO nanocomposites showed promising activity against under-investigated pathogens through mechanisms such as membrane disruption, apoptosis activation, and oxidative stress, which is caused by reactive oxygen species (ROS). Gram-positive bacteria, with their thick peptidoglycan layers, were more sensitive to these effects compared to the more complex outer membrane structure of Gram-negative bacteria, which resists ROS and ion penetration. The shape, size, and biocompatibility of CoO NPs also contributed to enhanced antibacterial activity by increasing bacterial membrane permeability and generating ROS that damage critical cellular components. These findings suggest that GPC films doped with CoO NPs have strong potential as effective antibacterial materials for food packaging applications. These findings suggest that GPC films doped with CoO NPs have strong potential as effective antibacterial materials for food packaging applications [27,28].

### Antioxidant activity

DPPH (1,1-diphenyl-2-picrylhydrazyl) is a relatively stable organic radical that has been used to evaluate the antioxidant activity of nanocomposites. Different concentrations (5, 10, 20, 40, 100, and 200 µg/mL) of nanocomposite films showed different percentages of scavenging activity, and results were compared with the reference, ascorbic acid. As the free radical’s scavenging activity is concentration dependent, interestingly, the scavenging activity of each film with or without CoO-NPs was increased with an increase in concentration. Similarly, the scavenging activity was also increased as the concentration of CoO-NPs increased. On the basis of results, these films are recognized for their ability to scavenge reactive oxygen species (ROS), such as singlet oxygen, superoxide anion, and hydroxyl radicals [26]. The minimum concentration of the nanocomposites required to inhibit 50% of the radical, the IC_50_ value, was calculated. As the IC_50_ value and antioxidant activity of test compounds are inversely proportional, the IC50 value of GPC/CoO nanocomposites was going to decrease as the concentration of CoO-NPs increased in GPC film. The IC_50_ values of the DPPH scavenging activity of GPC biocomposites components and the GPC/CoO nanocomposite are presented in Table 4.

**Table 4:** Antioxidant activity of CoO-NPs, GPC blend and GPC/CoO nanocomposites.

Compound	% Scavenging ± sd / Concentration (µg / mL)						IC_50_ µg/ mL
	200	100	40	20	10	5	
CoO-NPs	32 ± 1	26 ± 1	21 ± 1	15 ± 2	7 ± 1	1 ± 1	>300
GPC	52 ± 1	48 ± 1	36 ± 1	25 ± 1	16 ± 1	9 ± 1	>150
GPC + 0.5% CoO-NPs	58 ± 1	46 ± 2	31 ± 1	22 ± 1	14 ± 1	5 ± 1	146
GPC + 1.0% CoO-NPs	63 ± 1	54 ± 1	41 ± 1	29 ± 2	18 ± 1	11 ± 1	120
GPC + 1.5% CoO-NPs	63 ± 1	54 ± 2	42 ± 2	34 ± 2	23 ± 1	11 ± 1	116
GPC + 2.0% CoO-NPs	79 ± 2	66 ± 1	51 ± 1	38 ± 1	26 ± 2	14 ± 1	77
GPC + 2.5% CoO-NPs	88 ± 1	81 ± 1	77 ± 1	65 ± 1	38 ± 1	21 ± 1	18
Ascorbic acid	87 ± 0.5	84 ± 1	80 ± 0.25	70 ± 0.5	56 ± 1	35 ± 1	8.75 ± 0.5

### Antibiofilm activity

**Fig 7:**
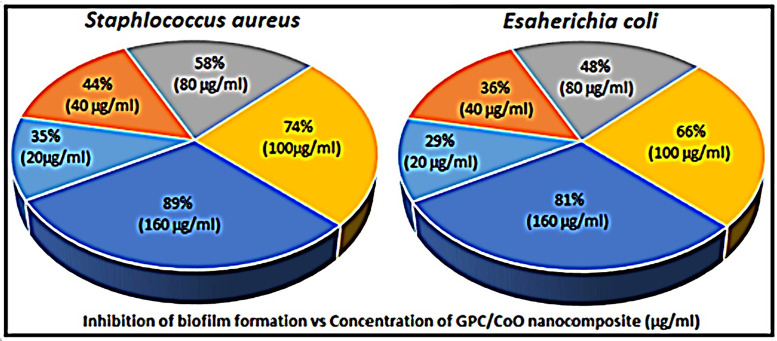
Biofilm inhibition activity of GPC/2.5% CoO-NPs in DMSO, *p* < 0.05.

The biofilm reduction ability of the Gelatin/ Polyethylene Glycol/ Chitosan (GPC) blend and GPC-CoO nanocomposite was evaluated against *S. aureus* and *E. coli* as model microorganisms and data presented in Fig 7. GPC with higher concentration of CoO-NPs (ca. 2.5%), exhibited higher antibiofilm activity as compared to neat GPC. At 160 µg/mL concentrations of composite materials, the percentages of biofilm reduction for *S. aureus* and *E. coli* were 89 and 81 respectively. It was due to Augmentation of GPC biocomposites films with CoO-NPs showed inhibition of biofilm formation more effectively [30].

## Conclusions

The research focuses on the preparation of Gelatin/ Polyethylene glycol/ Chitosan (GPC) nanocomposite films doped with CoO-NPs by solution casting technique followed by ultra-sonication method. Structural analysis by FT-IR, XRD and SEM, confirmed the successful incorporation of CoO-NPs in Gelatin/ Polyethylene glycol/ Chitosan (GPC) films, which improved the mechanical strength, flexibility, and thermal stability of the GPC films. Water resistance properties, including reduced permeability and solubility, were reduced up to 2.0% w/w addition of CoO-NPs in Gelatin/ Polyethylene glycol/ Chitosan blend. The GPC/CoO nanocomposites also showed significant antibacterial activity, against both Gram-positive and Gram-positive bacteria, due to the synergistic effects of gelatin, polyethylene and chitosan’s antimicrobial action and the membrane-disrupting properties of CoO NPs. Results showed that CoO improved the mechanical strength, thermal stability, and barrier properties of the gelatin/PEG/chitosan (GPC) films. It also provided higher antioxidant and UV-absorption ability, which can help preserve packaged food. CoO nanoparticles were easily synthesized and dispersed uniformly at low cost, making them a practical and effective alternative for active food packaging. These findings suggest that GPC doped with CoO-NPs is an excellent and sustainable option to maintain the quality and shelf-life for active food packaging.
